# Neighborhood differences in social capital in Ghent (Belgium): a multilevel approach

**DOI:** 10.1186/1476-072X-12-52

**Published:** 2013-11-13

**Authors:** Tijs Neutens, Veerle Vyncke, Dieter De Winter, Sara Willems

**Affiliations:** 1 Department of Geography, Ghent University, Ghent, Belgium; 2 Department of Family Medicine and Primary Health Care, Ghent University, Ghent, Belgium; 3 Research Foundation Flanders, Brussels, Belgium

## Abstract

**Background:**

Little research has focused on the spatial distribution of social capital, despite social capital’s rising popularity in health research and policy. This study examines the neighborhood differences in social capital and the determinants that explain these differences.

**Methods:**

Five components of neighborhood social capital are identified by means of factor and reliability analyses using data collected in the cross-sectional SWING study from 762 inhabitants in 42 neighbourhoods in the city of Ghent (Belgium). Neighborhood differences in social capital are explored using hierarchical linear models with cross-level interactions.

**Results:**

Significant neighborhood differences are found for social cohesion, informal social control and social support, but not for social leverage and generalized trust. Our findings suggest that neighborhood social capital depends on both characteristics of individuals living in the neighborhood (attachment to neighborhood) and characteristics of the neighborhood itself (deprivation and residential turnover). Our analysis further shows that neighborhood deprivation reinforces the negative effect of declining neighborhood attachment on social cohesion and informal social control.

**Conclusions:**

This study foregrounds the importance of contextual effects in encouraging neighborhood social capital. Given the importance of neighborhood-level characteristics, it can be anticipated social capital promoting initiatives are likely to be more effective when tailored to specific areas. Second, our analyses show that not all forms of social capital are influenced by contextual factors to the same extent, implying that changes in neighborhood characteristics are conducive to, say, trust while leaving social support unaffected. Finally, our analysis has demonstrated that complex interrelationships between individual- and neighborhood–level variables exist, which are often overlooked in current work.

## Background

### Neighborhood social capital and health

Recent years have witnessed a burgeoning academic interest in neighborhood effects on health [1,2]. Evidence is mounting that neighborhoods with a high level of poverty and unemployment have a greater incidence of health problems [3]. Living in a deprived area is associated with a shorter life expectancy [4,5], a greater presence of mental health problems [6,7] and worse self-rated health [8,9].

In an effort to explain health inequalities among neighborhoods, scholars have relied on the concept of neighborhood social capital [2,10,11]. Neighborhood social capital is found to be associated with mental health and reduce mortality [6,11-13] as well as detrimental health behaviors such as smoking and alcohol consumption [5,14-18]. Furthermore, research suggests that the positive association between neighborhood social capital and outcomes of wellbeing and health is stronger in deprived neighborhoods compared to non-deprived neighborhoods [11,19,20].

While social capital is generally seen as conducive for health, it can also negatively impact health and well-being [21,22]. Strong social bonds within a group may lead to closed social networks that exclude non-members, pushing them into an outsider role. Within these closed groups (e.g. a gang) undesirable norms can be maintained. Furthermore, high levels of social capital may exert social pressure on group members as a consequence of restricting social control and the need to conform to the prevailing social norms within the group [23].

### Carpiano’s model of social capital

Despite the relevance of neighborhood social capital for health, there is as yet no generally accepted definition of the concept. Nonetheless, various theoretical frameworks have been suggested in an attempt to provide useful conceptualizations of social capital (e.g., [2,10,24]). Most of these frameworks are inspired by the seminal work of Bourdieu [25] and/or Putnam [26,27].

A recently introduced framework of social capital is that of Carpiano [2] who defined social capital as the amount and type of resources that reside in social networks. The strength of Carpiano’s framework is that it focuses on neighborhood social capital and applies the social capital theory of Bourdieu [25] but also tries to integrate the Putnamesque tradition [26,27]. Following Bourdieu, Carpiano defines social capital as the resources present in social networks. However, he additionally acknowledges the importance of neighborhood social processes such as trust and reciprocity (which he labels as ‘social cohesion’), which are central to Putnam’s theory as the social processes that are needed to enable the exchange of social capital. Carpiano does so in order to address the criticism that research on social capital and health is undertheorized. Furthermore, since the framework includes individual as well as neighborhood characteristics, it lends itself well to determine to what extent neighborhood differences in social capital may be explained by compositional effects (e.g. gender composition within a neighborhood) or ‘true’ contextual effects (e.g. neighborhood deprivation). Carpiano [2] unfolds social capital into four components: (i) social support, (ii) social leverage, (iii) informal social control and (iv) neighborhood organization participation. Social support refers to a form of social capital that people can draw upon to cope with daily problems. Social leverage helps residents to access information and advance on the socio-economic ladder. Informal social control is the ability of residents to collectively maintain social order. Neighborhood organization participation refers to the ability of residents to organize collective activities to address neighborhood issues. In addition to these components, Carpiano includes social cohesion in the model as a distinct construct “because it represents networks and values from which social capital can be developed and used for action” ([2], p. 170). Finally, Carpiano [2] conceptualizes trust as a part of social cohesion, while other authors, among them Eriksson [24], see trust as an outcome of social capital. Social cohesion, generalized trust and the four forms of social capital can be examined separately to ascertain how spatial differences in each of these constructs can be explained by an interplay of compositional and contextual factors.

### Policy interest in social capital

Alongside academic interest, the favorable health implications of social capital have also not escaped policy makers’ attention. Specifically in Flanders, the regional government seeks to encourage social capital through the Pact 2020 strategy (equivalent to the European 2020 strategy), which includes among others improving community life by stimulating individuals in participating in different organizations. Investing in social capital is often considered as a strategy to promote public health and wellbeing (Vlaams Economisch Sociaal Overlegcomité, 2009) and the role of the regional level in this context is emphasized. However, policy makers should take into account the neighborhood differences in social capital across [24,28]. Since neighborhood social capital is shaped by natural, historical and cultural characteristics of neighborhoods, its relationship with health outcomes is expected to exhibit significant neighborhood differences. Therefore, the effectiveness of an intervention intended to foster social capital depends on the neighborhood where it is implemented [24,29].

### Neighborhood differences in social capital

Several studies describe significant regional differences in social capital, including Beugelsdijk & Van Schaik [30] and Van Oorschot, Arts & Gelissen [31]. Other studies were conducted at much smaller scales such as those focusing on the social capital difference between rural and urban areas [32-34] and between neighborhoods within the same city [35]. While insightful, most of these studies (ibid.) did not attempt to explain why spatial variations in social capital occur. However, a detailed insight in spatial variations of social capital and its determinants would contribute to the scarce theoretical basis on how social capital can be fostered. To that end, some scholars have recently sought to identify the socio-demographic and environmental characteristics of neighborhoods that are responsible for social capital formation.

Various studies (e.g. [2,36,37]) suggested that ethnicity is of major importance in the development of neighborhood social capital. People born in non-European countries appear to participate less in social activities and have a lower level of social capital than those born in Europe [37]. Further, the absence of trust in a neighborhood is also considered to be inimical to neighborliness and social vibrancy [2,26]. The absence of trust is influenced by individual factors such as age, ethnicity, being single and socio-economic status [36]. People who live in neighborhoods characterized by high-income inequality tend to exhibit low trust levels [38,39]. Furthermore, neighborhoods with a high percentage of elderly (65 years or older) tend to be associated with diminished levels of neighborhood social capital [36,38]. Another factor commonly associated with neighborhood social capital is physical disorder, being the level of physical stress a neighborhood suffers from as a consequence of, for example, littering, exhaust fumes, noise and odor nuisance. This association has been thoroughly discussed in Wilson and Kelling’s [40] ‘broken windows’ theory as well as in Skogan’s [41] ‘disorder and decline’ model. Generally, increasing levels of physical disorder are associated with declining levels of social cohesion and social control on deviants [42]. Finally, residential mobility is unfavorable to social capital formation as it inhibits the process of creating bonding and bridging ties [1,35]. Regarding environmental effects, neighborhood design and walkability are often put forward as being conducive to social fabric development [43,44]. This study seeks to integrate the above individual and neighborhood-level explanatory variables into one study and explore their effect on social capital.

### Research questions

Drawing on Carpiano’s model of social capital, this study uses multilevel modeling to investigate neighborhood differences in social capital in the city of Ghent (Belgium). The study explores whether social capital significantly differs between neighborhoods, and what determinants are associated to social capital at both the individual and neighborhood level. This research questions inherently refers to the debate of contextual and compositional influences of neighborhoods. Since it is very likely that both individual and neighborhood level variables influence the perception of neighborhood social capital, a simultaneous exploration of both compositional and contextual effects is needed. Multilevel modeling is the most appropriate analytical method to answer this research question. The outcome variable needs to be measured at the individual level to perform traditional multilevel analyses, which enables the identification of contextual and compositional sources of variation and accounts for the dependency in the data (i.e. individuals in neighborhoods) [45].

The paper has three specific research questions: (i) To what extent does social capital differ across neighborhoods in Ghent?; (ii) Can these neighborhood differences (partially) be explained by the characteristics of neighborhood inhabitants (compositional effect)?; (iii) Can the neighborhood differences in social capital (partially) be explained by neighborhood characteristics (contextual effect)? (iv) Does neighborhood context influence the association between social capital and its explanators?

In addressing these research questions, this paper adds to the knowledge base on neighborhood social capital in at least four important ways. First, while prior studies have largely considered social capital as a regressor for health outcomes, only few studies explicitly examined social capital as a regressand with individual and neighborhood characteristics as the regressors. Second, with some exceptions (e.g., [35]), research has primarily concentrated on regional, urban/rural and inter-city differences in social capital, while little attention has been paid to specific neighborhood differences within cities. Third, this study takes the theoretically underpinned and multidimensional framework of Carpiano [2] as a starting point and separately examines the different components that constitute neighborhood social capital. Fourth, multilevel analysis is used to investigate neighborhood differences in social capital and to disentangle compositional and contextual effects as has been called for in different studies [36,46-48]. Furthermore, this paper takes into account that previous research found a significant interaction between social capital and neigbhorhood deprivation [20,49], by actively exploring whether neighborhood context influences the assocation between social capital and it’s determinants.

## Methods

This study uses data gathered in the 2011–2014 Social capital and Well-being In Neighborhoods in Ghent (SWING) study. The SWING study provides information on social processes, health and socio-demographic characteristics in Ghent (Belgium) at both the individual and neighborhood level in three successive waves of data collection [50]. This study employs data of 762 neighborhood inhabitants in 42 neighborhoods in Ghent gathered during the second wave of data collection in 2012. The SWING survey uses a questionnaire, consisting of a face-to-face interview using a standardized questionnaire and a self-administered questionnaire. Sensitive questions such as questions on income, alcohol use and drug use are gathered through the self-administered questionnaire. This is done in order to minimize the risk of non-response. collected via face-to-face interviews. The response rate was 51%. The survey data is complemented with data from existing, external databases from the City of Ghent (available at http://gent.buurtmonitor.be) and Ghent University, containing mostly demographic and socio-economic data*.*

### Study setting and sampling procedure

**Figure 1:**
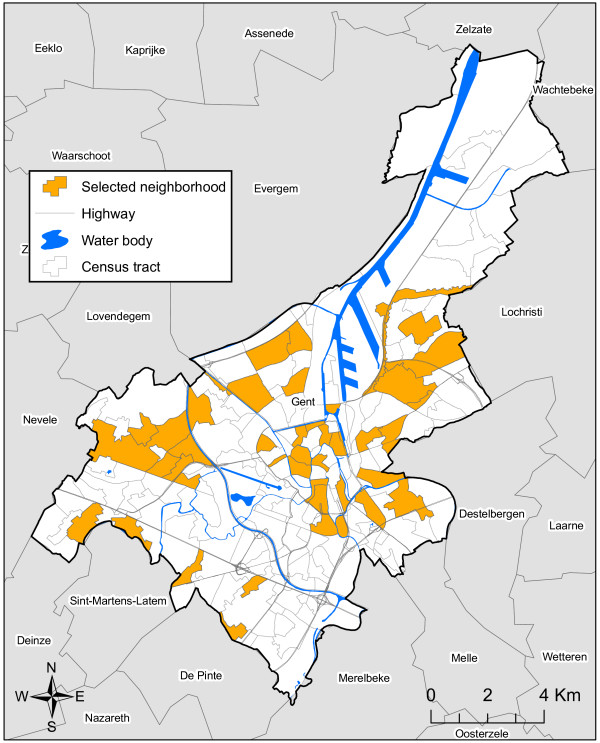
SWING-study wave 2: study area and selected neighborhoods.

Ghent is a medium-sized city in Belgium, 158 km^2^ in size, with approximately 250.000 inhabitants (1.506/km^2^). The city is divided into 201 statistical sectors. A statistical sector (henceforth referred to as ‘neighborhood’) comprises the smallest level at which demographic and socio-economic information is systematically gathered in Belgium and can be compared to the Anglo-Saxon census tract level. A sample of 42 neighborhoods has purposively been selected based on four criteria: (i) a minimum population size of 200 inhabitants; (ii) representativeness in terms of population density; (iii) representativeness in terms of deprivation level, based on the dynamic analysis of neighborhoods in difficulties by Vandermotten and colleagues [51]; and (iv) minimal inclusion of adjacent neighborhoods to avoid spatial autocorrelation. If bordering neighborhoods were selected, preference was given to neighborhoods separated by clear physical boundaries such as water bodies or highways. Figure 1 gives an overview of the selected neighborhoods in wave 2 of the SWING-study. Within each of the 42 selected neighborhoods a representative sample of inhabitants was selected from the municipal registry, stratified based on age, gender and nationality. For each selected inhabitant, three substitutes were selected within the same category with regard to age, gender and origin. Respondents who could not be reached or refused to participate were replaced by a randomly selected respondent from the corresponding age, gender and ethnic stratum, striving for a total of 20 inhabitants per neighbourhood. Persons who were younger than 18 years old at the time of the survey, those who had insufficient knowledge of the Dutch language and those who lived in a residential setting (e.g. home for the elderly, prison, etc.) were excluded from the survey [50].

In addition to the questionnaire for the inhabitants, information about neighbourhood social capital has also been obtained from key informants through a separate questionnaire. Key informants are people who work in one of the selected neighbourhoods and can observe what is happening in these neighbourhoods. They often have on average more knowledge about the social processes under study and can provide more useful and less biased information. Previous research has demonstrated that this method is able to create ecologically reliable and valid measures of neighbourhood social processes [50]. In total, 638 key informants were surveyed. It should be noted that the key informants have not been recruited from the set of inhabitants as to avoid bias in the level of neighborhood social capital. In other words, none of the 638 key informants is one of the 762 inhabitants.

### Dependent variables

The operationalization of the dependent variable, neighborhood social capital, is based on the multidimensional and theoretical framework provided by Carpiano [2]. The dependent variables include informal social control, social support, social leverage, social cohesion, and generalized trust in a neighborhood. Although neighborhood organization participation is included in the theoretical framework of Carpiano, this concept will not be considered in the analyses as no data was available on this component of social capital.

The components of neighborhood social capital are measured using a 4-point or 5-point Likert scale, except for generalized trust which is measured using a Visual Analogue Scale (VAS) between 0 and 10 (Table 1). Item responses were summed to create a single scale for each component of social capital, with a higher score referring to higher levels of social capital. The maximum score for each social capital component thus depends on the number of measurement items. The maximum score for social cohesion, informal social control, social support, social leverage and generalized trust are 20, 30, 20, 25 and 30, respectively. Results of the factor analyses (forced one-factor solutions in an exploratory principal axis factoring analysis) and reliability analyses used to construct the final scales are reported in Table 1. The social capital scales are all unidimensional, with acceptable to good internal consistency. Cronbach’s α ranges from 0.79 to 0.88, which are situated above the generally accepted cut-off values of 0.70-0.80.

**Table 1:** Five components of neighborhood social capital: overview of indicators and results of factor and reliability analyses

**Summative scale and individual items**	**Coding**	**Factor loadings (1) & Cronbach’s Alpha (2)**
***Social cohesion***		***0.83 (2)***
1. People around here are willing to help their neighbors	Strongly Agree → Strongly Disagree**	0.78 (1)
2. This is a close-knit neighborhood	Strongly Agree → Strongly Disagree**	0.71 (1)
3. People in this neighborhood can be trusted	Strongly Agree → Strongly Disagree**	0.69 (1)
4. Contacts between inhabitants in this neighborhood are generally positive	Strongly Agree → Strongly Disagree**	0.81 (1)
***Informal social control***		***0.87 (2)***
How likely is it that you could count on neighbors intervening when…		
1. Children were skipping school and hang out on a street corner	Very Likely → Very Unlikely**	0.69 (1)
2. Children were spray-painting graffiti on a local building	Very Likely → Very Unlikely**	0.74 (1)
3. Children were showing disrespect to an adult	Very Likely → Very Unlikely**	0.74 (1)
4. A fight breaks out in front of their house	Very Likely → Very Unlikely**	0.75 (1)
5. Children were making too much racket	Very Likely → Very Unlikely**	0.70 (1)
6. Children are using soft drugs (smoking weed, hasj, etc.)	Very Likely → Very Unlikely**	0.74 (1)
***Social support***		***0.79 (2)***
1. People in this neighborhood give or advice to each other (emotional/informational support).	Never → Often*	0.70 (1)
2. People in this neighborhood give material aid and assistance to each other (tangible support)	Never → Often*	0.80 (1)
3. People in this neighborhood show affection for each other (affectionate support).	Never → Often*	0.61 (1)
4. People in this neighborhood can call on each other to do enjoyable things (positive social interaction).	Never → Often*	0.68 (1)
***Social Leverage***		***0.88 (2)***
How often does it happen that people in this neighborhood give each other advice on…		
1. Child rearing	Never → Often*	0.71 (1)
2. Job openings	Never → Often*	0.80 (1)
3. Welfare and other benefits	Never → Often*	0.77 (1)
4. Education and courses	Never → Often*	0.81 (1)
5. Finances	Never → Often*	0.74 (1)
**Generalized Trust**		***0.76 (2)***
Most people in this neighborhood can be trusted	0 → 10	0.71 (1)
Most people in this neighborhood would try to take advantage of you	0 → 10	0.75 (1)
Most people in this neighborhood try to be helpful	0 → 10	0.68 (1)

Note: * 4- point Likert scale; **5- point Likert scale; original questionnaire in Dutch.

### Independent variables

Relevant independent variables at both the individual and neighborhood level are selected based on the relevant literature. At the individual level, this study takes the following variables into account: age, gender, ethnicity, having a partner and educational attainment. Each of these variables has been dichotomized. Age is dichotomized with persons younger than 65 years old being the reference category. This classification aligns with prior work [36,38], indicating that the 65+ age cohort tends to dispose of less social capital than their younger counterparts. The reference category for gender are men. Regarding ethnicity, respondents are considered to have a different ethnic background if one or both of the parents do not have the Belgian nationality (having the Belgian nationality served as the reference category). For ‘having a partner’, those with a partner served as the reference category. Educational attainment is dichotomized with a lower degree (degree up to the third year of secondary school) serving as the reference category. Since the correlation of educational attainment with income is higher (r = 0.350) than with other variables and income has a high number of missing values (n = 81), income has not been included as an explanatory variable.^a^ Finally, two dummy variables on residential length (with people living in the neighborhood for at least 5 years serving as the reference category) and neighborhood attachment (reference category: high neighborhood attachment) are included. It is hypothesized that a short residential stay (i.e. less than 5 years) negatively affects social capital at the individual level, while neighborhood attachment is assumed to positively influence the presence of neighborhood social capital (see also [2,24]). People were asked to report to what extent they feel attached to their neighborhood, using a 5-point Likert scale ranging from ‘strongly disagree’ to ‘strongly agree’. Those who agreed with feeling attached to their neighborhood served as the reference category.

At the neighborhood level, four variables are included in the analyses: neighborhood deprivation, percentage of elderly (65 years or older), residential mobility and physical disorder. To determine whether or not a neighborhood is deprived, a classification made by Vandermotten et al. [51] is used. These authors created an index which defines deprivation based on the accumulative presence of 22 unfavorable indicators related to factors such as income, education and housing. Non-deprived neighborhoods are considered as the reference category, since we are interested in the effect of socio-economic deprivation.

Since income and ethnicity are already implicitly covered by this multidimensional index of deprivation, these concepts are not separately included in the analyses for reasons of multicollinearity. Residential mobility of a neighborhood is measured by means of turnover (i.e. number of migration movements per 1000 inhabitants). It is hypothesized that high turnover erodes neighborhood social capital as it inhibits social ties to be adequately formed [1]. Although neighborhood design, measured in terms of land use mix and walkability, could have an effect on neighborhood social capital (see e.g., [43,44]), this variable highly correlated with turnover (r = 0.823) and is therefore not taken up in the statistical analysis. To account for the age composition of neighborhoods, the percentage of elderly in the neighborhood is included to determine whether a neighborhood with a high level of people aged 65+ tends to dispose of less social capital (reference category: people younger than 65). Physical disorder was represented by a four-item Likert scale. Key informants were asked (on a five-point scale) how often they have observed each of the following four occurrences in their neighborhood: (1) ‘litter on the streets’, (2) ‘exhaust gases’, (3) ‘noise pollution’, and (4) ‘bad smell’. The scale has an alpha of 0.74. The scale is constructed following a two-step procedure ([52]). First, summative scales were calculated at the individual (key-informant) level. Consequently, these individual scores were aggregated to the neighborhood level. This variable is the only one that uses the data provided by the key informants.

### Analysis

SPSS Statistics 21 is used for data preparation and exploratory analyses. To assess the geographical variation of neighborhood social capital, multilevel linear regression analyses are fitted using maximum likelihood estimation in MLWIN 2.26. Multilevel analysis accounts for the nested data structure of people within neighborhoods and allows for estimation of (i) the effect of individual and neighborhood level factors on neighborhood social capital (fixed part) and (ii) the variation in social capital among neighborhoods that cannot be accounted for by the included predictors (random part) [11,36,47]. Furthermore, multilevel modeling enables insight into the extent to which potential neighborhood differences in social capital are due to either individual-level characteristics (compositional variation) or characteristics of the neighborhoods themselves (contextual variation) [36,47]. Due to a high correlation between the different components of neighborhood social capital (ranging from 0.158 to 0.625) and to enable a differentiated analysis, models were fitted for each social capital component separately.

A three-step sequential strategy is used to run the multilevel models. First, a null model (model 0) is fitted, without any level 1 or level 2 predictors. This model serves as a benchmark to which the other models are compared, following the difference in deviance test. Model 1a includes only individual-level variables to determine to what extent differences in neighborhood social capital can be explained as a compositional effect. Model 2a additionally includes neighborhood variables and identifies the neighborhood-level variables that explain the geographic variation of neighborhood social capital. Parsimonious models, that contain only the individual and neighborhood predictors that are significantly associated with neighborhood social capital, are composed in order to maximize statistical power (models 1b and 2b). To estimate whether the explanatory variables have a different effect in different neighborhood settings, models that allow for a random slope of the significant predictor variables are explored. Finally, a cross-level interaction is modeled to study the interaction between neighborhood deprivation and variables influencing neighborhood social capital. Since some research suggests an interaction between neighborhood social capital and deprivation, we want to explore if neighborhood deprivation affects the relationship between social capital and the independent variables.

## Results

The characteristics of the 762 respondents are listed in Table 2. These characteristics closely mirror those of the actual population of Ghent (see Hardyns et al., [50] for a statistical analysis of the representativeness of the sample).

**Table 2:** Sample characteristics

	**N**	**%**	**m**	**sd**	**Range (Min – Max)**
***Individual level***					
*Age (in years)*			48.65	19.02	77 (18–95)
*65 years or older*	180	21.3%			
*Male*	370	48.6%			
*Female*	392	51.4%			
*Single*	199	26.1%			
*Not Belgian*	91	11.9%			
*Low education level*	130	17.1%			
*Short residential stay (5 years or less)*	226	29.7%			
***Neighborhood level***					
*Deprivation*	9	21.5%			
*Turnover (per 1000 inhabitants)*	42		241.32	136.64	469.51 (94.05-564.01)
*Physical disorder*	42		14.31	2.71	11.25 (8.75-20)

N = absolute number, m = mean, sd = standard deviation.

**Figure 2:**
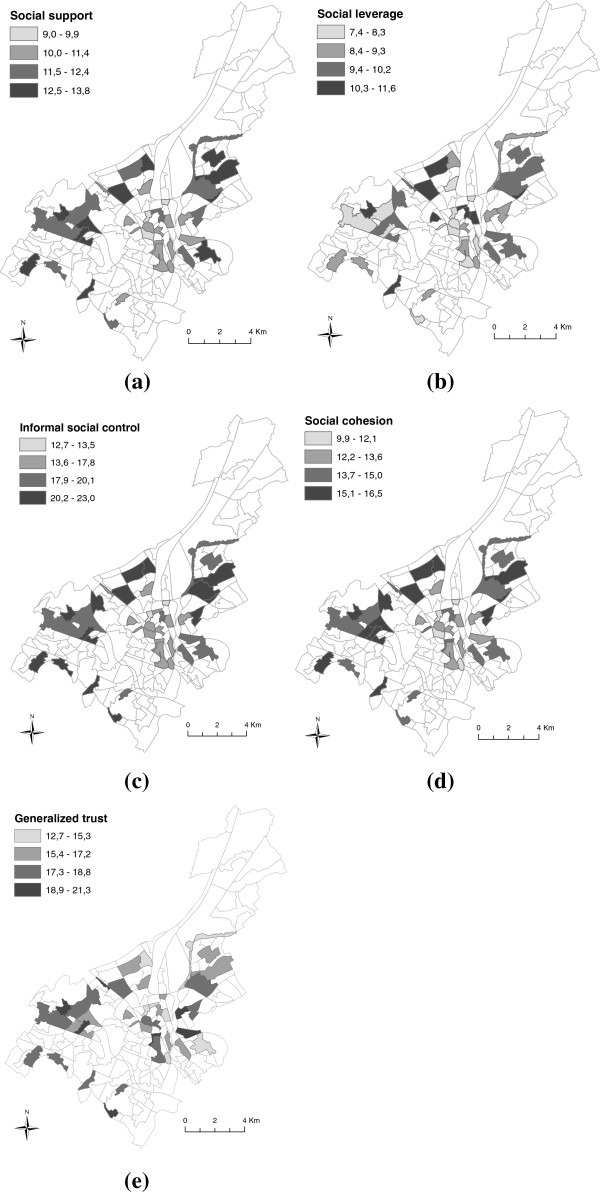
Differences in the neighborhood average of individual scores on the scales of (a) social support, (b) social leverage, (c) informal social support, (d) social cohesion and (e) generalized trust.

Figure 2 illustrates neighborhood differences for each of the five social capital scales using Jenks’ [53] natural breaks classification. To create this figure, the summative scales which reflect inhabitants perceptions on neighborhood social capital are aggregated to obtain a neighborhood level score for each of the components of social capital. The neighborhood averages equal 14.2 (±1.4, [9.9,16.6]), 18.9 (±2.4, [12.7,23.0]), 11.7 (±1.1, [9,13.8]), 9.3 (±1.2, [11.6,7.4]) and 17.3 (±1.9, [12.7,21.3]) for social cohesion, informal social control, social support, social leverage and generalized trust, respectively.

The results of the multilevel models are summarized below. Each component of neighborhood social capital is reported separately in Tables 3, 4, 5, 6 and 7. All social capital components significantly differ between neighborhoods, exept for social leverage. However, adding neighborhood determinants in the analyses nullifies the signficant neighborhood variation in social capital.The intraclass correlation coefficient (ICC) in the null model for the different components of neighborhood social capital ranges from 4.6 (social leverage) to 16.1% (social cohesion), which suggest that a modest part of the variation in neighborhood social capital can be attributed to the neighborhood level.

**Table 3:** Fixed and random parameters of the social cohesion multilevel models

	**Model 0**	**Model 1a**	**Model 1b**	**Model 2a**	**Model 2b**
	β (SE)	β (SE)	β (SE)	β (SE)	β (SE)
*Fixed effects*					
Constant	14.241 (0.215)***	14.903 (0.246)***	14.920 (0.199)***	15.038 (0.219)***	15.121 (0.170)***
*Level 1*					
Female		−0.031 (0.199)		−0.034 (0.198)	
65 years or older		0.086 (0.257)		0.050 (0.256)	
Low education level		−0.001 (0.288)		0.058 (0.283)	
Single		−0.572 (0.231)*	−0.527 (0.228)*	−0.493 (0.230)*	−0.491 (0.226)*
Not Belgian		0.076 (0.329)		0.276 (0.329)	
Weak neighborhood attachment		−1.941 (0.236)***	−1.962 (0.233)***	−1.891 (0.233)***	−1.901 (0.230)***
<5 years of residence		−0.009 (0.236)		0.076 (0.235)	
*Level 2*					
Deprived neighborhood				−0.960 (0.337)*	−0.977 (0.351)**
Percentage of elderly				−0.156 (0.142)	
Turnover				−0.552 (0.173)***	−0.557 (0.143)***
Physical disorder				−0.188 (0.177)	
*Random effects*					
*Level 1*					
Constant	7.852 (0.414)***	7.244 (0.384)***	7.246 (0.383)***	7.232 (0.383)***	7.253 (0.383)***
*Level 2*					
Constant	1.510 (0.425)***	0.964 (0.300)***	0.944 (0.295)***	0.247 (0.143)	0.298 (0.154)
Intraclass correlation	0.161	0.117	0.115	0.033	0.039
Log likelihood	3795,593	3688,729	3714,429	3656,402	3685,974
∆ Log likelihood (∆ df)		106,864	81,164	139,191	109,619
*P*		< 0.001	< 0.001	< 0.001	< 0.001

Note: *p < 0.05, **p < 0.01, ***p < 0.001.

Model 0: null model without level 1 and level 2 variables; Model 1a: model with only level 1 variables; Model 1b: parsimonious model with only level 1 variables; Model 2a: model with level 1 and level 2 variables; Model 2b: parsimonious model with level 1 and level 2 variables.

**Table 4:** Fixed and random parameters of the informal social control multilevel models

	**Model 0**	**Model 1a**	**Model 1b**	**Model 2a**	**Model 2b**
	β (SE)	β (SE)	β (SE)	β (SE)	β (SE)
*Fixed effects*					
Constant	18.883 (0.369)***	19.341 (0.463)***	19.358 (0.360)***	19.569 (0.401)***	19.719 (0.283)***
*Level 1*					
Female		0.268 (0.370)		0.246 (0.368)	
65 years or older		−0.805 (0.476)		−0.884 (0.475)	
Low education level		0.385 (0.536)		0.330 (0.526)	
Single		0.283 (0.430)		0.429 (0.427)	
Not Belgian		−0.894 (0.610)		−0.461 (0.610)	
Weak neighborhood attachment		−1.858 (0.439)***	−1.725 (0.434)***	−1.775 (0.432)***	−1.631 (0.428)***
<5 years of residence		0.267 (0.439)		0.397 (0.436)	
*Level 2*					
Deprived neighborhood				−1.479 (0.681)*	−1.644 (0.624)*
Percentage of elderly				−0.268 (0.256)	
Turnover				−1.236 (0.312)***	−1.224 (0.254)***
Physical disorder				−0.329 (0.318)	
*Random effects*					
*Level 1*					
Constant	25.541 (1.348)***	24.885 (1.319)***	25.277 (1.336)***	6.667 (0.344)***	25.263 (1.335)***
*Level 2*					
Constant	4.277 (1.256)***	3.551 (1.083)***	3.429 (1.057)***	0.000 (0.000)	0.804 (0.487)
Intraclass correlation	0.143	0.125	0.119	0.000	0.031
Log likelihood	4671,624	4603,412	4651,175	4567,579	4617,973
∆ Log likelihood (∆ df)		68,212	20,449	104,045	53,651
*P*		< 0.001	< 0.001	< 0.001	< 0.001

Note: *p < 0.05, **p < 0.01, ***p < 0.001.

Model 0: null model without level 1 and level 2 variables; Model 1a: model with only level 1 variables; Model 1b: parsimonious model with only level 1 variables; Model 2a: model with level 1 and level 2 variables; Model 2b: parsimonious model with level 1 and level 2 variables.

**Table 5:** Fixed and random parameters of the social support multilevel models

	**Model 0**	**Model 1a**	**Model 1b**	**Model 2a**	**Model 2b**
	β (SE)	β (SE)	β (SE)	β (SE)	β (SE)
*Fixed effects*					
Constant	11.691 (0.169)***	12.465 (0.207)***	12.317 (0.160)***	12.382 (0.190)***	12.193 (0.117)***
*Level 1*					
Female		−0.069 (0.191)		−0.064 (0.190)	
65 years or older		−0.183 (0.246)		−0.197 (0.244)	
Low education level		−0.102 (0.273)		−0.052 (0.266)	
Single		−0.462 (0.221)*	−0.464 (0.217)*	−0.350 (0.219)	
Not Belgian		0.153 (0.312)		0.336 (0.313)	
Weak neighborhood attachment		−1.816 (0.224)***	−1.839 (0.220)***	−1.769 (0.219)***	−1.787 (0.216)***
<5 years of residence		−0.276 (0.224)		−0.149 (0.223)	
*Level 2*					
Deprived neighborhood				−0.115 (0.288)	
Percentage of elderly				−0.175 (0.107)	
Turnover				−0.567 (0.133)**	−0.618 (0.102)***
Physical disorder				−0.194 (0.134)	
*Random effects*					
*Level 1*					
Constant	7.163 (0.378)***	6.707 (0.355)***	6.688 (0.353)***	6.677 (0.344)***	6.719 (0.354)***
*Level 2*					
Constant	0.794 (0.261)**	0.381 (0.166)*	0.410 (0.172)*	0.000 (0.000)	0.055 (0.095)
Intraclass correlation	0.100	0.054	0.058	0.000	0.008
Log likelihood	3708,957	3608,963	3632,345	3576,124	3615,077
∆ Log likelihood (∆ df)		99,994	76,612	132,833	93,880
*P*		< 0.001	< 0.001	< 0.001	< 0.001

Note: *p < 0.05, **p < 0.01, ***p < 0.001.

Model 0: null model without level 1 and level 2 variables; Model 1a: model with only level 1 variables; Model 1b: parsimonious model with only level 1 variables; Model 2a: model with level 1 and level 2 variables; Model 2b: parsimonious model with level 1 and level 2 variables.

**Table 6:** Fixed and random parameters of the social leverage multilevel models

	**Model 0**	**Model 1a**	**Model 1b**	**Model 2a**	**Model 2b**
	β (SE)	β (SE)	β (SE)	β (SE)	β (SE)
*Fixed effects*					
Constant	9.291 (0.178)***	10.116 (0.261)***	9.972 (0.194)***	9.918 (0.265)***	9.969 (0.189)***
*Level 1*					
Female		−0.348 (0.257)		−0.348 (0.257)	
65 years or older		−0.743 (0.331)*	−0.905 (0.304)**	−0.679 (0.333)	
Low education level		−0.525 (0.364)		−0.579 (0.363)	
Single		0.087 (0.297)		0.196 (0.297)	
Not Belgian		0.559 (0.416)		0.605 (0.424)	
Weak neighborhood attachment		−1.736 (0.298)***	−1.775 (0.296)***	−1.717 (0.300)***	−1.716 (0.297)***
<5 years of residence		−0.013 (0.299)		0.100 (0.302)	
*Level 2*					
Deprived neighborhood				0.557 (0.424)	
Percentage of elderly				−0.292 (0.158)	
Turnover				−0.418 (0.195)*	−0.262 (0.154)
Physical disorder				−0.150 (0.197)	
*Random effects*					
*Level 1*					
Constant	12.649 (0.672)***	11.975 (0.639)***	12.057 (0.642)***	11.975 (0.639)***	12.056 (0.642)***
*Level 2*					
Constant	0.612 (0.291)*	0.329 (0.222)	0.367 (0.231)	0.130 (0.179)	0.300 (0.216)
Intraclass correlation	0.046	0.027	0.030	0.011	0.019
Log likelihood	4057,65	3969,854	4003,094	3960,668	4000,306
∆ Log likelihood (∆ df)		87,796	54,556	96,982	57,344
*P*		< 0.001	< 0.001	< 0.001	< 0.001

Note: *p < 0.05, **p < 0.01, ***p < 0.001.

Model 0: null model without level 1 and level 2 variables; Model 1a: model with only level 1 variables; Model 1b: parsimonious model with only level 1 variables; Model 2a: model with level 1 and level 2 variables; Model 2b: parsimonious model with level 1 and level 2 variables.

**Table 7:** Fixed and random parameters of the generalized trust multilevel models

	**Model 0**	**Model 1a**	**Model 1b**	**Model 2a**	**Model 2b**
	β (SE)	β (SE)	β (SE)	β (SE)	β (SE)
*Fixed effects*					
Constant	17.263 (0.292)***	18.220 (0.427)***	18.072 (0.318)***	18.418 (0.450)***	
*Level 1*					
Female		−0.336 (0.400)		−0.333 (0.400)	
65 years or older		0.440 (0.514)		0.402 (0.517)	
Low education level		−1.356 (0.570)*	−1.173 (0.541)*	−1.275 (0.573)*	
Single		−0.425 (0.461)		−0.435 (0.463)	
Not Belgian		0.330 (0.652)		0.487 (0.664)	
Weak neighborhood attachment		−2.219 (0.466)***	−2.234 (0.459)***	−2.125 (0.471)***	
<5 years of residence		0.050 (0.468)		0.034 (0.474)	
*Level 2*					
Deprived neighborhood				−1.123 (0.791)	
Percentage of elderly				0.100 (0.299)	
Turnover				0.158 (0.363)	
Physical disorder				−0.018 (0.372)	
*Random effects*					
*Level 1*					
Constant	30.262 (1.596)***	29.312 (1.553)***	29.441 (1.557)***	29.319 (1.554)***	
*Level 2*					
Constant	1.888 (0.783)*	1.454 (0.679)*	1.563 (0.703)*	1.244 (0.663)	
Intraclass correlation	0.059	0.047	0.050	0.041	
Log likelihood	4786,232	4713,448	4736,855	4710,668	
∆ Log likelihood (∆ df)		72,784	49,377	75,564	
*P*		< 0.001	< 0.001	< 0.001	

Note: *p < 0.05, **p < 0.01, ***p < 0.001.

Model 0: null model without level 1 and level 2 variables; Model 1a: model with only level 1 variables; Model 1b: parsimonious model with only level 1 variables; Model 2a: model with level 1 and level 2 variables; Model 2b: parsimonious model with level 1 and level 2 variables.

### Individual variables and social capital

The first model analyses the association of different individual characteristics with neighborhood social capital. Having a weak neighborhood attachment is associated with lower levels of all components of neighborhood social capital. Also, being single is associated with lower levels of social cohesion and social support. The 65+ cohort has more difficulty in using social leverage relative to their younger counterparts. Finally, results show that a low educational attainment contributes to lower trust levels. The association between these individual predictors and neighborhood social capital remains significant when neighborhood variables are accounted for (model 2a and 2b). The only exceptions are the relationship between age and social leverage and the relationship between being single and social support. Gender and short residential stay are not related to the presence of neighborhood social capital. Finally, none of the random slope models show a significant slope variance, they will therefore not be discussed. Results of these models are available from the author upon request.

### Neighborhood variables and social capital

Results show that neighborhood deprivation is associated with lower levels of neighborhood social cohesion and informal social control. Furthermore, having a high residential turnover at the neighborhood level is negatively related to all components of neighborhood social capital after taking the socio-demographic composition of the neighborhood into account, except for generalized trust. Also, after controlling for compositional effects, neighborhoods with high levels of turnover tend to exhibit less neighborhood social capital, except in the form of generalized trust. None of the neighborhood variables are significantly associated to levels of generalized trust, while neighborhood turnover is only marginally significantly associated to neighborhood social leverage.

### Cross-level interactions

Finally, cross-level interactions were examined between neighborhood deprivation and the significant individual determinants of neighborhood social capital. A significant interaction effect between neighborhood deprivation and neighborhood attachment was identified for social cohesion and for informal social control (β = −1.41, p = 0.003 and β = −2.37, p = 0.007). These results indicate that living in a deprived neighborhood reinforces the negative effect of declining neighborhood attachment on social cohesion and informal social control. None of the other variables show significant interaction effects.

## Discussion

The analysis shows that most of the researched components of social capital significantly vary across neighborhoods in Ghent. Overall, the contextual effect is modest but persistent after adjusting the null model for individual characteristics. In the variance components models, the ICC was highest for social cohesion, informal social control and social support. This shows that the proportion of the variance in neighborhood social capital, which can be attributed to the neighborhood level, is largest for these components of social capital. These findings align with a recent study by Baum et al. [54] who focused on health differences between neighborhoods and found significant between-neighborhood variation in social cohesion. They contend that social cohesion and informal social control are often linked together in the sense that communities with a strong sense of social cohesion are more able to exert informal social control to establish and maintain norms and reduce crime.

A clear difference is found between the different components of neighborhood social capital. Social cohesion and informal social control are affected by both individual and neighborhood aspects (contextual effect above a compositional effect), while social leverage and generalized trust are only explained by individual aspects (compositional effect). These findings suggest that the variation of social capital across neighborhoods can be seen as a contextual effect, especially for social cohesion and informal social control, but that individual-level predictors remain significant. By not including the relevant individual predictors, we could misinterpret the fixed part of a two-level statistical model [55].

At the individual level, the strongest predictor for neighborhood social capital is the level of neighborhood attachment. Weak neighborhood attachment is associated with lower levels of neighborhood social capital and this holds for all aspects of social capital. Low levels of neighborhood attachment disconnect people from networks that possess beneficial resources and provide social support in times of hardship [24]. Further, being single is significantly related with perceptions of neighborhood social support. This tends to suggest that people with a partner have more social ties and better access to social networks through their partner. This finding echoes that of Subramanian, Lochner and Kawachi [36] who observed that divorced people experience lower levels of social capital. Additionally, low educational attainment is associated with lower levels of generalized trust and people aged 65+ report lower levels of social leverage.

At the neighborhood level, neighborhood deprivation and residential turnover are the strongest predictors for neighborhood social capital. Residential turnover was significant for all but one component of social capital (i.e. generalized trust). The importance of residential turnover has also been highlighted in other research [1,35]. Creating social ties and forming social capital takes time and high residential mobility within a neighborhood strongly inhibits this process [1]. An additional contextual explanatory variable for social cohesion and informal social control is neighborhood deprivation. A possible explanation is that a deprived neighborhood setting has downward leveling norms and, consequently, a higher incidence of crime [56]. People feel less safe, resulting in a less socially cohesive neighborhood and the absence of norms and values that give rise to informal social control [43,54]. As opposed to other studies that found age distribution to be associated with associational involvement [38] and trust [36], age distribution, measured in terms of the percentage of elderly within a neighborhood, could only be associated with social cohesion but not with other components of neighborhood social capital.

Finally, we found interaction effects between neighborhood deprivation and neighborhood social processes. Previous research suggested that social processes in the neighborhood have a stronger health effect in deprived neighborhoods [11,20]. In this study, the interaction between neighborhood attachment and neighborhood deprivation is analyzed. For both social cohesion and informal social control, there was a significant interaction between neighborhood deprivation and neighborhood attachment. Social cohesion and informal social control tend to decline more strongly with decreasing levels of neighborhood attachment for those in deprived neighborhoods than for those in non-deprived neighborhoods.

### Limitations

Apart from the strengths summarized in the introduction, the study also has limitations, opening up avenues for further work. First, the compositional and contextual factors found to be responsible for generating spatial disparities in social capital are specific to the study area at hand and cannot be straightforwardly generalized to other urban contexts in Europe and beyond. Additional empirical evidence, especially from countries with sharper social inequalities, is desirable to help refine our insights into the factors and their interactions that steer the spatial development of neighborhood social capital [24]. Second, the current analysis is static and does not allow examining how social capital evolves over time. Comparing spatial differences of social capital at different points in time would enable evaluating before-after scenarios so as to directly measure the impact of territorial policy initiatives. To this end, researchers may rely on multilevel analyses for repeated measures in time, which will require the estimation of changing neighborhood effects while controlling for changing population composition. Third, because the data used are cross-sectional, reverse causation cannot be excluded. The relationship could go from neighborhood social capital to individual and neighborhood variables instead of vice versa [11]. For instance, self-selection may arise in the sense that deprived people are condemned to neighborhoods with low social capital rather than low social capital fostering neighborhood deprivation. Another data limitation is that people who do not speak Dutch have been excluded from the survey. This might have biased the relationship between ethnicity and social capital. Finally, when interpreting the results the reader should be alerted that there is an overlap related to content between neighborhood attachment and each of the social capital components.

### Relevance for researchers and practitioners in other contexts

While our results are specific to the current case study, they do have wider repercussions for policy strategies seeking to foster neighborhood social capital in other contexts than ours for at least three reasons. First, our study has foregrounded the importance of contextual effects in encouraging neighborhood social capital at the city scale. Furthermore, given the intermediating role of neighborhood-level characteristics, it can be anticipated that not all areas within a city will be equally responsive to social capital promoting initiatives. Such initiatives are likely to be more effective when tailored to specific areas. Second, our analysis further shows that not all forms of social capital are influenced by contextual factors to the same extent, implying that changes in neighborhood characteristics are conducive to, say, trust while leaving social support unaffected. Finally, our analysis has demonstrated that complex interrelationships between individual and neighborhood variables exist, which are often overlooked in current work. In particular, neighborhood deprivation seems to amplify or dampen the association between characteristics of neighborhood inhabitants and neighborhood social capital.

## Conclusions

This paper demonstrates that social capital significantly differs across neighborhoods in Ghent. Neighborhood factors partly explain this variation, after individual-level variables are controlled for. This study adds to the modest knowledge base concerning neighborhood differences in social capital. Our findings suggest that health-promoting initiatives to improve social capital should consider neighborhood attachment, neighborhood deprivation and residential turnover. Policy initiatives focussing on these aspects are likely to render neighborhood social capital more equitable across urban regions.

## Endnotes

^a^We have run multilevel models with and without individual income as an explanatory variable. Individual income is positively associated with generalized trust (p < 0.001) but exhibits no significant relationship with the other components of social capital. Adding individual income to the models did impact the estimation of the parameters for the independent variables (with the largest impact on the estimation of the parameters for age and nationality), but did not alter the findings described in this paper. The analyses including individual income are available from the author upon request.

## Competing interests

The authors declare that they do not have competing interests.

## Authors’ contributions

TN and VV performed the statistical analyses. DDW, TN and VV drafted the manuscript. SW coordinated the study. All authors reviewed the manuscript and approved the final version.
